# A new essential oil from the native Ecuadorian species *Steiractinia sodiroi* (Hieron.) S.F. Blake (Asteraceae): chemical and enantioselective analyses

**DOI:** 10.1038/s41598-023-44524-6

**Published:** 2023-10-11

**Authors:** Yessenia E. Maldonado, Omar Malagón, Nixon Cumbicus, Gianluca Gilardoni

**Affiliations:** 1 Departamento de Química, Universidad Técnica Particular de Loja (UTPL), Calle Marcelino Champagnat s/n, 110107 Loja, Ecuador; 2 Departamento de Ciencias Biológicas y Agropecuarias, Universidad Técnica Particular de Loja (UTPL), Calle Marcelino Champagnat s/n, 110107 Loja, Ecuador

**Keywords:** Secondary metabolism, Natural products

## Abstract

In the present study, the essential oil from dry leaves of *Steiractinia sodiroi* (Hieron.) S.F. Blake is described for the first time. The plant material, collected in the Province of Loja (Ecuador), was analytically steam-distilled in a Marcusson-type apparatus, affording an essential oil with a 0.2 ± 0.12% yield. The volatile fraction was submitted to GC–MS and GC–FID analyses, on two stationary phases of different polarity. A total of sixty-seven compounds, corresponding to 95.6–91.2% by weight of the whole oil mass, on the two columns respectively, were detected and quantified with at least one column. The quantification was carried out calculating the relative response factors of each constituent according to their combustion enthalpy. The major components were limonene (25.6–24.9%), sabinene (11.7–12.4%), germacrene D (7.7–7.0%), α-pinene (7.8–6.9%), δ-cadinene (7.3–7.0%), (*E*)-β-caryophyllene (4.8–4.5%), and bicyclogermacrene (3.6–3.0%). The chemical composition was complemented with the enantioselective analysis of some major chiral compounds, conducted by means of two β-cyclodextrin-based capillary columns. Three constituents, (*S*)-(+)-α-phellandrene, (*R*)-(−)-1-octen-3-ol, and (*S*)-(−)-limonene were enantiomerically pure, whereas (1*R*,5*R*)-(+)-β-pinene, (1*S*,5*S*)-(−)-sabinene, (*R*)-(−)-terpinen-4-ol, (*R*)-(+)-α-terpineol, and (*R*)-(+)-germacrene D presented an enantiomeric excess. Finally, α-pinene was present as a racemic mixture.

## Introduction

Ecuador is an Andean country, located at the equatorial latitude of the South American Pacific coast. Due to its orography and geographic location, it is characterized by four very different climatic regions, corresponding to the Galapagos Islands, the Coast, the Andes, and the Amazon rainforest. Thanks to these features, Ecuador is mentioned among the seventeen “megadiverse countries”, identified for possessing most of the vegetal and animal biodiversity in the world^1^. This fact converted Ecuador into an invaluable reserve of unprecedented botanical species, whose metabolic profile is still unknown^2,3^. For these reasons, during the last twenty years, our group have been involved in the search for new secondary metabolites in plants from the Ecuadorian flora and, in more recent years, in the chemical, enantioselective, and olfactometric description of novel essential oils^4–7^. In particular, the family Asteraceae demonstrated to be a very promising taxon for the study of volatile fractions, which chemical composition is often dominated by sesquiterpenes. In this context, the Andean native species *Steiractinia sodiroi* (Hieron.) S.F. Blake has been selected and the essential oil (EO), obtained from its leaves, was analysed and described here for the first time. The genus *Steiractinia* S.F. Blake is a small taxon of twenty-six species, of which only fifteen are accepted^8^. All the accepted species are mainly described in Colombia, except for *S. sodiroi* which stands as the only taxon of this genus present in Ecuador^9^. According to literature, *S. sodiroi* is a shrub, treelet or tree, growing in the provinces of Bolívar, Cañar, Chimborazo, Guayas, Imbabura, Loja, Pichincha, and Tungurahua, in the range of 0–3000 m above the sea level^10^. This species is also known with the following synonyms: *Aspilia sodiroi* Hieron., *Steiractinia grandiceps* S.F. Blake, *Steiractinia mollis* S.F. Blake, and *Steiractinia rosei* S.F. Blake^9^. So far, to the best of the authors’ knowledge, only three scientific papers have been published about the phytochemistry of the genus *Steiractinia.* On the one hand, two articles were published by Bohlmann et al. on the sesquiterpene lactones produced by *S. sodiroi* itself, including the first description of stereactinolides; whereas a more recent article was published by Gamboa-Carvajal et al. about the biological activity of hydro-ethanolic extracts from *S. aspera*^11–13^. On the other hand, only one article has recently been published about a volatile fraction from the genus *Steiractinia*, citing the chemical composition and biological activities of *S. aspera* EO^14^. Therefore, the present study constitutes the first investigation about the chemical and enantiomeric composition of an EO from *S. sodiroi*.

## Results

### Chemical analysis

**Figure 1:**
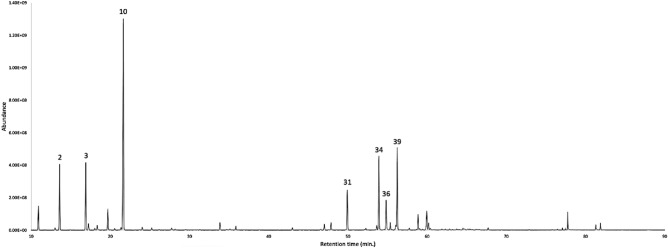
GC–MS profile of *S. sodiroi* EO on a 5%-phenyl-methylpolysiloxane stationary phase. The peak numbers refer to Table 1.

**Figure 2:**
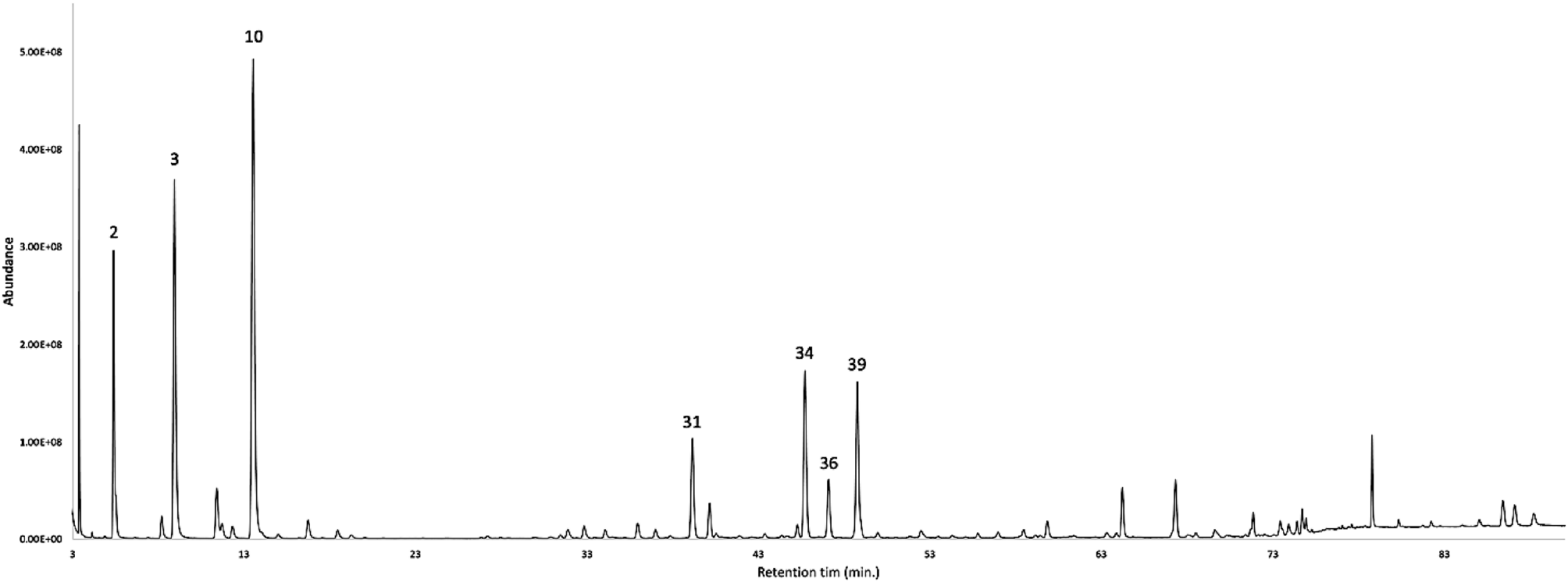
GC–MS profile of *S. sodiroi* EO on a polyethylene glycol stationary phase. The peak numbers refer to Table 1.

**Figure 3:**
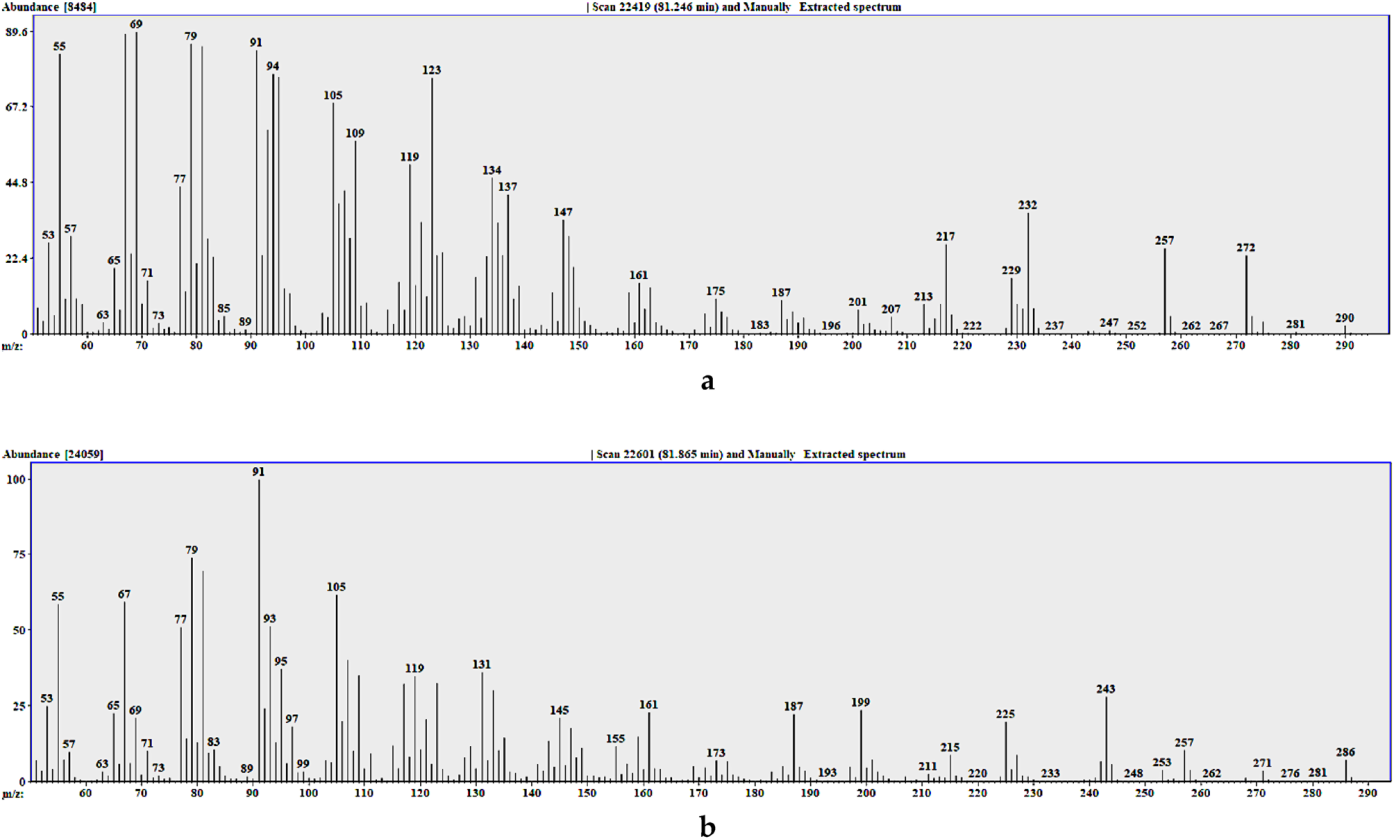
EIMS mass spectra of the undetermined compounds 65 (**a**) and 67 (**b**).

The dry leaves of *S. sodiroi* afforded, after steam-distillation, an EO with an estimated yield of 0.2 ± 0.12% as an analytical mean value over four repetitions. The EO, analysed on two stationary phases of different polarity, permitted to detect sixty-seven compounds, that were quantified on at least one column. The volatile fraction was dominated by monoterpene hydrocarbons, corresponding to 95.6–91.2% by weight of the whole oil mass, on a non-polar and polar column respectively. After that, the sesquiterpene hydrocarbons represented the second main fraction, with a content of 26.9–24.0% on the two columns. The major components of the EO (≥ 3.0% with at least one column) were limonene (25.6–24.9%), sabinene (11.7–12.4%), germacrene D (7.7–7.0%), α-pinene (7.8–6.9%), δ-cadinene (7.3–7.0%), (*E*)-β-caryophyllene (4.8–4.5%), and bicyclogermacrene (3.6–3.0%). The gas chromatographic profile with both stationary phases is represented in Figs. 1 and 2, whereas the complete chemical analysis is reported in Table 1. The mass spectra of the undetermined compounds are represented in Fig. 3.

**Table 1:** Qualitative (GC–MS) and quantitative (GC–FID) chemical composition of *S. sodiroi* EO on 5%-phenyl-methylpolysiloxane and polyethylene glycol stationary phases.

N°	Compound	5%-phenyl-methylpolysiloxane					polyethylene glycol				
		LRI^a^	LRI^b^	%	σ	References	LRI^a^	LRI^b^	%	σ	References
1	α-thujene	924	924	0.5	0.20	^15^	1020	1020	0.6	0.16	^16^
2	α-pinene	931	932	7.8	0.03	^15^	1015	1015	6.9	0.43	^17^
3	sabinene	972	969	11.7	4.68	^15^	1114	1115	12.4	3.49	^18^
4	β-pinene	976	974	1.0	0.12	^15^	1103	1103	0.9	0.08	^19^
5	1-octen-3-ol	986	974	0.2	0.07	^15^	1451	1451	0.3	0.06	^20^
6	myrcene	990	988	0.7	0.05	^15^	1159	1159	0.7	0.06	^21^
7	α-phellandrene	1007	1002	2.5	0.06	^15^	1154	1154	1.8	0.51	^22^
8	α-terpinene	1017	1014	0.4	0.21	^15^	1169	1169	0.5	0.18	^23^
9	*p*-cymene	1027	1020	0.4	0.02	^15^	1261	1261	0.3	0.07	^24^
10	limonene	1031	1024	25.6	1.65	^15^	1189	1189	24.9	2.47	^25^
11	γ-terpinene	1060	1054	0.6	0.32	^15^	1236	1236	0.8	0.31	^26^
12	*cis*-sabinene hydrate	1074	1065	0.4	0.04	^15^	1458	1459	0.6	0.16	^27^
13	terpinolene	1086	1086	0.2	0.06	^15^	1272	1271	0.2	0.06	^28^
14	*trans-*sabinene hydrate	1106	1098	0.5	0.10	^15^	1538	1538	0.5	0.15	^29^
15	*n*-nonanal	1111	1100	0.2	0.01	^15^	1387	1387	0.1	0.01	^30^
16	*trans*-*p*-mentha-2,8-dien-1-ol	1128	1119	0.1	0.03	^15^	1618	1618	0.2	0.03	^31^
17	*cis*-limonene oxide	1138	1132	0.1	0.01	^15^	1429	1430	trace	–	^32^
18	*trans*-verbenol	1152	1140	0.1	0.01	^15^	1665	1665	0.1	0.01	^33^
19	terpinen-4-ol	1185	1174	1.2	0.32	^15^	1589	1589	1.6	0.52	^34^
20	α-terpineol	1202	1186	0.3	0.07	^15^	1686	1687	0.2	0.03	^34^
21	decanal	1212	1201	0.6	0.05	^15^	1490	1490	0.4	0.06	^35^
22	undecanal	1314	1305	0.3	0.01	^15^	1595	1593	0.1	0.01	^36^
23	myrtenyl acetate	1328	1324	0.1	0.01	^15^	1673	1677	trace	–	^37^
24	hexyl tiglate	1335	1330	trace	–	^15^	1613	1621	trace	–	^38^
25	α-cubebene	1345	1348	0.1	0.01	^15^	1443	1443	trace	–	^39^
26	α-ylangene	1374	1373	0.7	0.09	^15^	1472	1472	0.6	0.07	^40^
27	β-bourbonene	1382	1387	trace	–	^15^	1497	1499	trace	–	^41^
28	geranyl acetate	1383	1379	trace	–	^15^	–	–	–	–	–
29	β-cubebene	1387	1387	0.9	0.16	^15^	1521	1521	0.7	0.13	^42^
30	*n*-tetradecane	1400	1400	trace	–	^15^	1400	1400	trace	–	–
31	(*E*)-β-caryophyllene	1419	1417	4.8	0.21	^15^	1573	1574	4.5	0.60	^43^
32	α-humulene	1456	1452	0.3	0.02	^15^	1644	1644	0.3	0.02	^44^
33	γ-curcumene	1478	1481	0.5	0.03	^15^	1677	1680	0.6	0.05	^37^
34	germacrene D	1482	1480	7.7	0.52	^15^	1684	1684	7.0	2.44	^45^
35	(*E*)-β-ionone	1484	1487	trace	–	^15^	1915	1915	trace	–	^46^
36	bicyclogermacrene	1496	1500	3.6	0.02	^15^	1708	1706	3.0	0.02	^47^
37	(*E*,*E*)-α-farnesene	1505	1505	0.7	0.20	^15^	1742	1740	overlapped to N°40		^48^
38	tridecanal	1517	1509	0.5	0.04	^15^	1805	1805	0.5	0.03	^49^
39	δ-cadinene	1520	1522	7.3	1.10	^15^	1738	1738	7.0	1.20	^50^
40	α-calacorene	1545	1544	0.3	0.03	^15^	1891	1893	0.3	0.04	^51^
41	(*E*)-nerolidol	1564	1561	2.0	0.02	^15^	2035	2036	1.9	0.22	^52^
42	β-calacorene	1568	1564			^15^	1933	1942	trace	–	^53^
43	1α,10α-epoxy-amorph-4-ene	1574	1570	0.1	0.03	^15^	–	–	–	–	–
44	spathulenol	1583	1577	2.7	0.08	^15^	2099	2098	2.3	0.14	^54^
45	caryophyllene oxide	1587	1582	1.2	0.02	^15^	1939	1938	1.0	0.01	^55^
46	α-corocalene	1623	1622	0.2	0.02	^15^	2037	2037	trace	–	^56^
47	muurola-4,10(14)-dien-1-β-ol	1633	1630	0.2	0.05	^15^	2127	§	0.2	0.02	–
48	α-acorenol	1633	1632			^15^	–	–	–	–	–
49	caryophylla-4(12),8(13)-dien-5α-ol	1645	1639	trace	–	^15^	2280	2285	trace	–	^37^
50	α-muurolol (= Torreyol)	1649	1644	0.4	0.24	^15^	2153	2155	0.4	0.14	^57^
51	α-cadinol	1663	1652	0.6	0.22	^15^	2210	2210	0.6	0.16	^51^
52	*cis*-calamenen-10-ol	1666	1660	0.2	0.02	^15^	2330	§	0.2	0.03	–
53	*trans*-calamenen-10-ol	1674	1668	0.3	0.11	^15^	2364	§	0.6	0.10	–
54	khusinol	1679	1679	0.3	0.02	^15^	2202	§	0.2	0.21	–
55	cadalene	1681	1675			^15^	2194	2196	trace	–	^58^
56	germacra-4(15),5,10(14)-trien-1-α-ol	1693	1685	0.4	0.19	^15^	2350	§	0.5	0.13	–
57	eudesma-4(15),7-dien-1β-ol	1696	1687			^15^	2331	2333	trace	–	^56^
58	amorpha-4,9-dien-2-ol	1699	1700	0.3	0.08	^15^	2278	§	0.2	0.05	–
59	pentadecanal	1722	1724	0.3	0.02	^59^	2017	2016	0.3	0.04	^60^
60	eicosane	1995	2000	0.1	0.01	^15^	2000	2000	trace	–	–
61	(*E*,*E*)-geranyl linalool	2025	2026	trace	–	^15^	2536	2537	trace	–	^61^
62	kaurene	2070	2042	0.2	0.01	^15^	2354	2351	0.2	0.05	^62^
63	heneicosane	2100	2100	0.1	0.07	^15^	2100	2100	0.1	0.01	–
64	phytol	2111	2111	1.5	0.40	^63^	2613	2611	1.8	0.42	^64^
65	undetermined (MW = 290)	2298	–	0.7	0.19	^15^	3030	–	1.0	0.20	–
66	tricosane	2300	2300			^15^	2300	2300	trace	–	–
67	undetermined (MW = 286)	2323	–	1.0	0.23	^15^	2986	–	1.3	0.30	–
	Monoterpene hydrocarbons			51.4					49.8		
	Oxygenated monoterpenes			2.7					3.2		
	Sesquiterpene hydrocarbon			26.9					24.0		
	Oxygenated sesquiterpenes			8.9					8.1		
	Diterpene hydrocarbons			0.2					0.2		
	Oxygenated diterpenes			3.3					4.1		
	Others			2.3					1.8		
	Total			95.6					91.2		

^a^Calculated linear retention index.

^b^Reference linear retention index, trace =  < 0.1% % = percent by weight, σ = standard deviation, MW = molecular weight, § = identified only by mass spectrum in the polar column.

### Enantioselective analysis

The enantioselective analysis permitted to investigate the enantiomeric excesses of nine chiral compounds inside *S. sodiroi* EO. Three of them, specifically (*S*)-(+)-α-phellandrene, (*R*)-(−)-1-octen-3-ol, and (*S*)-(−)-limonene, were present as enantiomerically pure substances, whereas α-pinene was observed as a racemic mixture. All the other chiral components appeared as scalemic mixtures. Almost all the chiral terpenes were analysed on a 2,3-diacetyl-6-*tert*-butyldimethylsilyl-β-cyclodextrin chiral selector, with the exception of limonene and germacrene D, that were submitted to separation on 2,3-diethyl-6-*tert*-butyldimethylsilyl-β-cyclodextrin since they are inseparable with the other chiral selector. The detailed results of the enantioselective analysis are exposed in Table 2.

**Table 2:** Enantioselective analysis of some chiral terpenes from *S. sodiroi* EO.

Enantiomer	LRI	Distribution (%)	*e.e*. (%)
(1*S*,5*S*)-(−)-α-pinene	925*	50.1	0.2
(1*R*,5*R*)-(+)-α-pinene	926*	49.9	
(1*S*,5*S*)-(−)-β-pinene	978*	32.9	34.1
(1*R*,5*R*)-(+)-β-pinene	979*	67.1	
(1*R*,5*R*)-(+)-sabinene	1007*	5.1	89.9
(1*S*,5*S*)-(−)-sabinene	1011*	94.9	
(*S*)-(+)-α-phellandrene	1025*	100.0	100.0
(*R*)-(−)-1-octen-3-ol	1229*	100.0	100.0
(*R*)-(−)-terpinen-4-ol	1338*	73.5	47.0
(*S*)-(+)-terpinen-4-ol	1379*	26.5	
(*S*)-(−)-α-terpineol	1402*	37.6	24.7
(*R*)-(+)-α-terpineol	1407*	62.4	
(*S*)-(−)-limonene	1049**	100.0	100.0
(*R*)-(+)-germacrene D	1502**	98.2	96.3
(*S*)-(−)-germacrene D	1509**	1.8	

*LRI* linear retention index, *e.e.* enantiomeric excess.

*2,3-diacetyl-6-*tert*-butyldimethylsilyl-β-cyclodextrin.

**2,3-diethyl-6-*tert*-butyldimethylsilyl-β-cyclodextrin column.

## Discussion

The chemical analysis was carried out on two columns with stationary phases of different polarity, that afforded reciprocally consistent results from both the qualitative and quantitative point of view. The main components are some common mono and sesquiterpenes, with limonene as the dominant constituent accounting for about one quarter of the whole oil. According to the chemical composition, some biological activities could be expected. In particular, due to the very high abundance of limonene (more than one quarter of the total composition), the anti-inflammatory, antioxidant, anticancer, antinociceptive, and antidiabetic activities should be considered as suitable to be investigated in further studies^65^.

**Figure 4:**
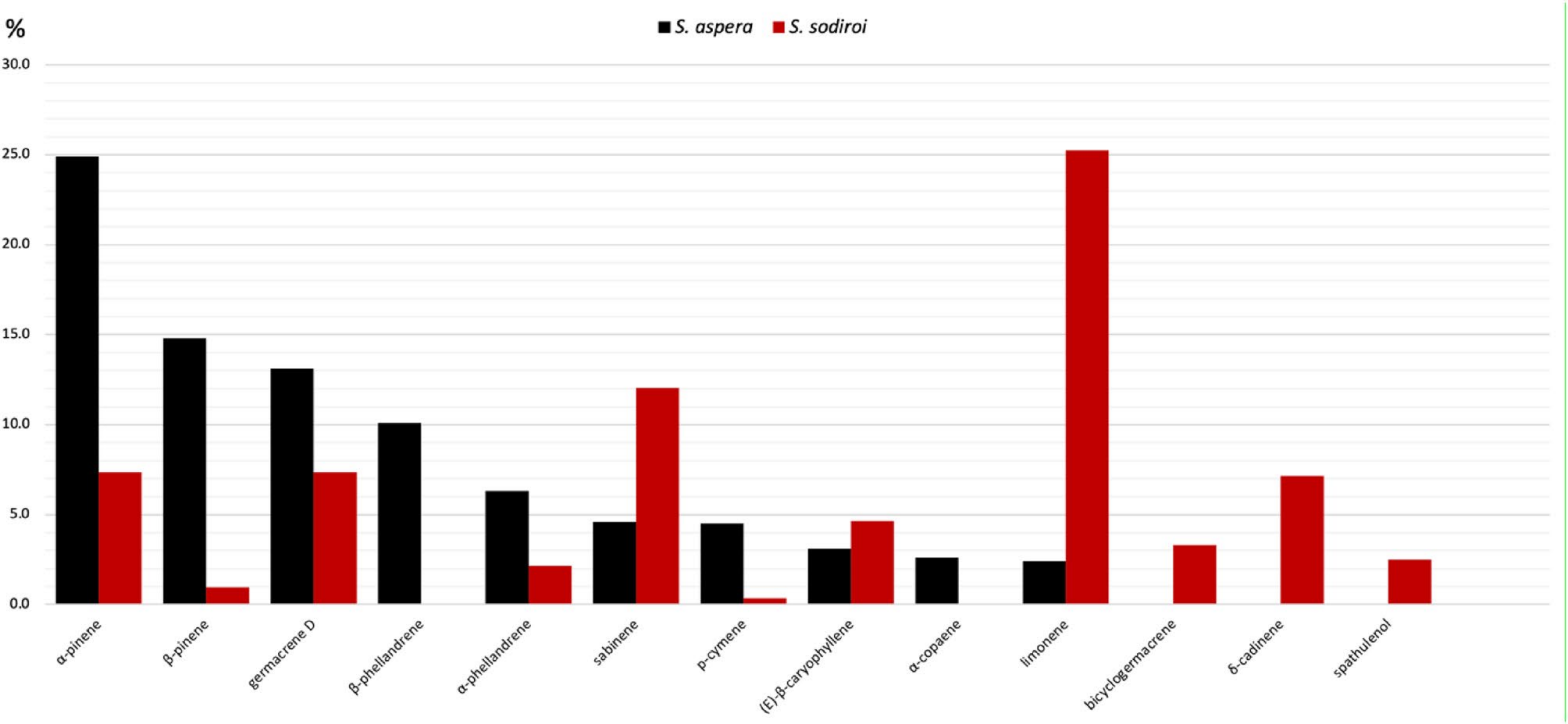
Compared abundance of major EO components of *S. aspera* and *S. sodiroi.*

About the chemical composition, the only EO described in literature from the genus *Steiractinia* is the one obtained from *S. aspera*^14^. The comparison between the major component abundance of the two volatile fractions is represented in Fig. 4. As it can be observed, both EOs are dominated by some common monoterpenes and many of them are shared by both volatile fractions. However, despite the similar qualitative composition, the relative abundances are quite different. In fact, in *S. aspera* EO, the very major component is α-pinene, whereas *S. sodiroi* EO is dominated by limonene (both about 25%). Furthermore, of the other *S. aspera* important components, β-phellandrene and α-copaene are absent in *S. sodiroi* whereas, on the other hand, bicyclogermacrene, δ-cadinene, and spathulenol are absent or minority compounds in *S. aspera* EO.

**Figure 5:**
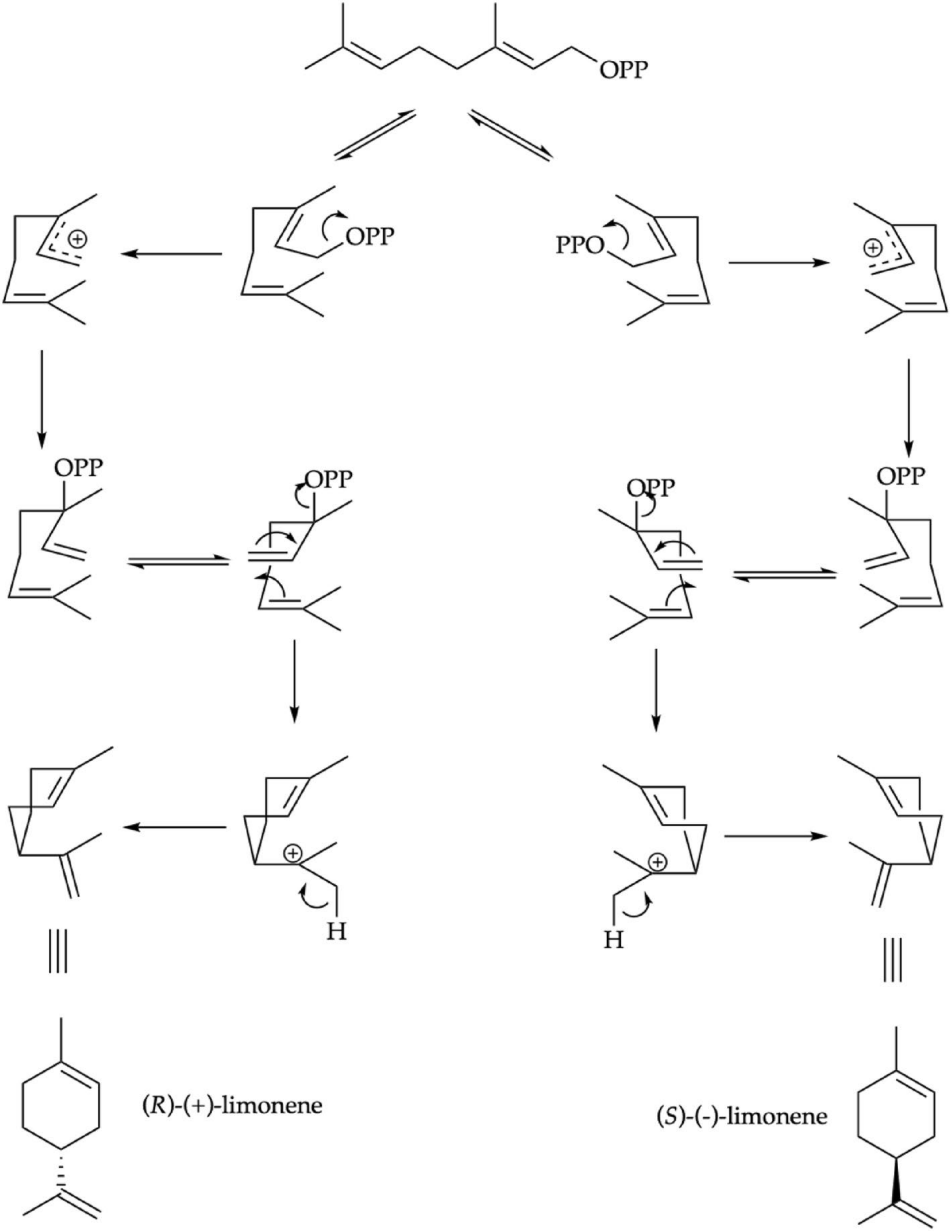
Possible mechanism for the enantioselective biosynthesis of limonene.

The enantioselective analyses showed that some chiral components were enantiomerically pure, others were present as scalemic mixtures, whereas α-pinene was detected as a racemic mixture. These results demonstrated that, as usual, different enantiomers are produced by *S. sodiroi* metabolism, with the aim of carrying on different biological functions. The enantiomeric excess of a chiral compound in nature can be explained with the enantiospecific kinetic resolution of a racemic mixture or because of enzymatic enantioselective reactions on a non-chiral precursor. The latter case is represented in Fig. 5, where each enantiomer of limonene can be obtained from a specific conformer of the non-chiral precursor geranyl pyrophosphate, enzymatically stabilized respect to the other one. It is well known that the enantiomers of the same metabolite are often characterized by enantioselective biological properties such as, for example, different aromas^66^. These differences, mainly due to the chiral character of receptors and enzymes, often determine enantioselective biological activities. In the present EO, the dominant component is present in an enantiomerically pure form, being (*S*)-(−)-limonene the only detected enantiomer. According to literature, the laevorotatory form of limonene is sometimes more active than the dextrorotatory one as an antibacterial agent, for instance against *Staphylococcus aureus*, *Escherichia coli*, *Klebsiella pneumoniae*, *Moraxella catarrhalis*, and *Cryptococcus neoformans*^67^.

## Methods

### Plant material

The leaves of *S. sodiroi* were collected on 24 September 2020, on the slopes of Mount Villonaco, Province of Loja, from different shrubs located in the range of 500 m around a central point of coordinates 04° 00′ 01″ S and 79° 15′ 23″ W, at 2548 m above the sea level. The collection was conducted under permission of the Ministry of Environment, Water and Ecological Transition of Ecuador, with MAATE registry number MAE-DNB-CM-2016-0048. The identification of the botanical species was carried out by one of the authors (N.C.), according to botanical specimens with code 3319939 conserved at the National Museum of Natural History, Smithsonian Institution, Washington, DC, USA. A voucher was deposited at the herbarium of the Universidad Técnica Particular de Loja with code 14666. The plant material was dried the same day of collection, at 35 °C for 48 h, and stored in a fresh, dark place until use.

### Distillation and sample preparation

Four amounts (50 g, 35 g, 35 g, and 35 g respectively) of dry leaves were separately steam-distilled for four hours, in the Marcusson-type apparatus previously described in literature^68^. The distillation was conducted analytically, over 2 mL of cyclohexane spiked with *n*-nonane as internal standard (0.69 mg/mL). After distillation, the EO solutions were recovered, stored in the dark at − 14 °C and directly injected into GC for the analyses. The solvent and internal standard were purchased by Sigma-Aldrich (St. Louis, MO, USA).

### Qualitative and quantitative chemical analyses

The qualitative chemical analyses were conducted through gas chromatography–mass spectrometry (GC–MS), in a Trace 1310 gas chromatograph, coupled to a simple quadrupole detector model ISQ 7000 (Thermo Fisher Scientific, Walthan, MA, USA). The MS was operated in SCAN mode, with a mass range of 40–400 m*/*z. The electron ionization (EI) ion source was set at 70 eV, whereas both ion source and transfer line were programmed at the temperature of 230 °C. The injections were repeated in two columns with stationary phases of different polarity, a DB-5 ms and a HP-INNOWax, both purchased from Agilent Technology (Santa Clara, CA, USA). The stationary phases were based on 5%-phenyl-methylpolysiloxane and polyethylene glycol respectively, whereas the columns were 30 m long and characterized by 0.25 mm internal diameter and 0.25 μm film thickness. The oven was programmed according to the following thermal gradient, that was applied to both columns: 50 °C for 5 min, followed by a ramp of 3 °C/min until 100 °C, then a second ramp of 5 °C/min until 180 °C, and finally 10 °C/min until 230 °C. The final temperature was maintained for 10 min. The injector was operated in split mode, set at 230 °C, and injecting 1 μL of EO solution with a split ratio of 40:1. The carrier gas was GC grade helium, set at the constant flow of 1 mL/min, and purchased from Indura, Guayaquil, Ecuador. All the EO components were identified comparing the respective linear retention index and mass spectra with data from literature (see Table 1). The linear retention indices (LRIs) were calculated according to Van den Dool and Kratz, with respect to a series of homologous *n*-alkanes in the range C_9_–C_22_^69^. All the alkanes were purchased from Sigma-Aldrich.

The quantitative analyses were carried out with the same GC, columns, configuration, and thermal program of the qualitative ones, except for the use of a flame ionization detector (FID) instead of MS and the value of split ratio (10:1). All the detected compounds were quantified calculating each relative response factor (RRF) versus isopropyl caproate, according to their combustion enthalpy^70,71^. A six-point calibration curve was built for each column, using isopropyl caproate as calibration standard and *n*-nonane as internal standard, according to what was previously described in literature^72^. Both curves produced a correlation coefficient of 0.998. The internal standard was purchased from Sigma-Aldrich, whereas isopropyl caproate was synthetised in the authors’ laboratory and purified until 98.8% (GC–FID purity).

### Enantioselective analysis

The *S. sodiroi* EO was also submitted to enantioselective analysis, through two enantioselective capillary columns, based on 2,3-diacetyl-6-*tert*-butyldimethylsilyl-β-cyclodextrin and 2,3-diethyl-6-*tert*-butyldimethylsilyl-β-cyclodextrin respectively. Both columns were 25 m long, 250 μm internal diameter and 0.25 μm phase thickness, purchased from Mega, MI, Italy. The analysis was conducted in the same GC–MS instrument used for the qualitative ones, with the following thermal program: 50 °C for 1 min, a thermal gradient of 2 °C/min until 220 °C, that were maintained for 10 min (total time 96 min). The carrier gas (He) was set at the constant pressure of 70 kPa. Sample volume, split ratio, injector temperature, transfer line temperature, and MS parameters were the same as the qualitative analyses. The enantiomers were identified by means of the respective MS spectra and linear retention indices, and through the injection of enantiomerically pure standards, that were purchased from Sigma-Aldrich.

### Policy statement about plant investigation

The authors declare that all experimental research and field studies on plants (either cultivated or wild), including the collection of plant material, were carried out in accordance with relevant institutional, national, and international guidelines and legislation. The species *Steiractinia sodiroi* (Hieron.) S.F. Blake appears neither in the IUCN Red List of Threatened Plants nor in the Red Book of the Endemic Plants of Ecuador. Furthermore, this study was conducted under permission of the Ministry of Environment, Water and Ecological Transition of Ecuador, with MAATE registry number MAE-DNB-CM-2016-0048. Finally, the authors declare that a minimal quantity of plant material was collected to carry on the present investigation, avoiding any injury to the shrubs at the collection site.

## Conclusions

The leaves of *Steiractinia sodiroi* (Hieron.) S.F. Blake produce an EO with about 0.2% distillation yield respect to the dry plant material. This EO is rich in monoterpene hydrocarbons, being limonene the main component (about 25%). The enantioselective analysis demonstrated the presence of some enantiomerically pure chiral terpenes, as well as scalemic and racemic mixtures of other compounds. The major constituent, limonene, was enantiomerically pure as a laevorotatory isomer. As usual, this feature demonstrated that different enantiomers are produced by *S. sodiroi* metabolism, through enantioselective biosynthetic steps or enantiospecific kinetic resolution of some chiral intermediates.

## Data Availability

The datasets used and/or analysed during the current study available from the corresponding author on reasonable request.
